# Chemical Profiling and Antioxidant, Antimicrobial, and Hemolytic Properties of *Euphorbia calyptrata* (l.) Essential oils: *in Vitro* and *in Silico* Analysis

**DOI:** 10.1002/open.202300243

**Published:** 2024-03-25

**Authors:** Fatima El Kamari, Otmane Zouirech, Amira Metouekel, Mohammed Bouslamti, Imane Maliki, Abdelfattah El Moussaoui, Mohamed Chebaibi, Mohamed Taibi, Abdulaziz Abdullah Alsahli, Hiba‐Allah Nafidi, Mohammed Bourhia, Musaab Dauelbait, Abdelfattah Abdellaoui

**Affiliations:** ^1^ Laboratory of Engineering, Electrochemistry, Modeling and Environment Faculty of Sciences Dhar El Mahraz Sidi Mohammed Ben Abdellah University, B. P. 1796 Fes-Atlas Morocco; ^2^ Laboratory of Natural Substances, Pharmacology, Environment, Modeling, Health and Quality of Life Faculty of Sciences Dhar El Mahraz University Sidi Mohamed Ben Abdellah Fez Morocco; ^3^ Euromed Research Center Euromed Faculty of Pharmacy Euromed University of Fez Fez 30000 Morocco; ^4^ Laboratory of Health and Environment Department of Biology Moulay Ismail University Meknes 50050 Morocco; ^5^ Plant Biotechnology Team Faculty of Sciences Abdelmalek Essaadi University Tetouan 93002 Morocco; ^6^ Biomedical and Translational Research Laboratory Faculty of Medicine and Pharmacy of Fez University of Sidi Mohamed Ben Abdellah Fez 30070 Morocco; ^7^ Ministry of Health and Social Protection Higher Institute of Nursing Professions and Health Techniques Fez Morocco; ^8^ Laboratoire d'Amélioration des Productions Agricoles, Biotechnologie et Environnement (LAPABE) Faculté des Sciences Université Mohammed Premier Oujda 60000 Morocco; ^9^ Department of Botany and Microbiology College of Science King Saud University P. O. BOX 2455 Riyadh 11451 Saudi Arabia; ^10^ Department of Food Science Faculty of Agricultural and Food Sciences Laval University, 2325 Quebec City QC G1V 0A6 Canada; ^11^ Laboratory of Biotechnology and Natural Resources Valorization Faculty of Sciences Ibn Zohr University Agadir 80060 Morocco; ^12^ Department of Scientific Translation Faculty of Translation University of Bahri Khartoum 11111 Sudan

**Keywords:** *Euphorbia*, GC-MS, Antioxidant, Hemolytic, NADPH, Antibacterial, Antifungal

## Abstract

In this work, we sought to validate the use of *Euphorbia calyptrata* (L.), a Saharan and Mediterranean medicinal plant, in traditional pharmacopeia. GC‐MS/MS identified volatile compounds of potential therapeutic interest. Antioxidant tests were performed using β‐carotene decolorization, DPPH radical scavenging, FRAP, beta‐carotene bleaching, and TAC. The antimicrobial activity was evaluated on solid and liquid media for bacterial and fungal strains to determine the zone of inhibition and the minimum growth concentration (MIC) of the microbes tested. The hemolytic activity of these essential oils was assessed on red blood cells isolated from rat blood. Phytochemical characterization of the terpenic compounds by GC‐MS/MS revealed 31 compounds, with alpha‐Pinene dominating (35.96 %). The antioxidant power of the essential oils tested revealed an IC_50_ of 67.28 μg/mL (DPPH), EC_50_ of 80.25.08±1.42 μg/mL (FRAP), 94.83±2.11 μg/mL (beta carotene) and 985.07±0.70 μg/mL (TAC). Evaluating solid media‘s antibacterial and antifungal properties revealed a zone of inhibition between 10.28 mm and 25.80 mm and 31.48 and 34.21 mm, respectively. On liquid media, the MIC ranged from 10.27 μg/mL to 24.91 μg/mL for bacterial strains and from 9.32 μg/mL to 19.08 μg/mL for fungal strains. In molecular docking analysis, the compounds naphthalene, shogunal, and manol oxide showed the greatest activity against NADPH oxidase, with Glide G scores of −5.294, −5.218 and −5.161 kcal/mol, respectively. For antibacterial activity against *E. coli* beta‐ketoacyl‐[acyl carrier protein] synthase, the most potent molecules were cis‐Calamenene, alpha.‐Muurolene and Terpineol, with Glide G‐scores of −6.804, −6.424 and −6.313 kcal/mol, respectively. Hemolytic activity revealed a final inhibition of 9.42±0.33 % for a 100 μg/mL concentration. The essential oils tested have good antioxidant, antimicrobial, and hemolytic properties thanks to their rich phytochemical composition, and molecular docking analysis confirmed their biological potency.

## Graphical abstract

*Euphorbia calyptrata* (L.) is a medicinal plant used in traditional medicine. In the present work, essential oils from *Euphorbia calyptrate* were studied in terms of chemical composition, antioxidant, antimicrobial, and hemolytic properties. Results showed that *Euphorbia calyptrate* essential oils possessed promising aforementioned activities thanks to their richness in bioactive compounds.

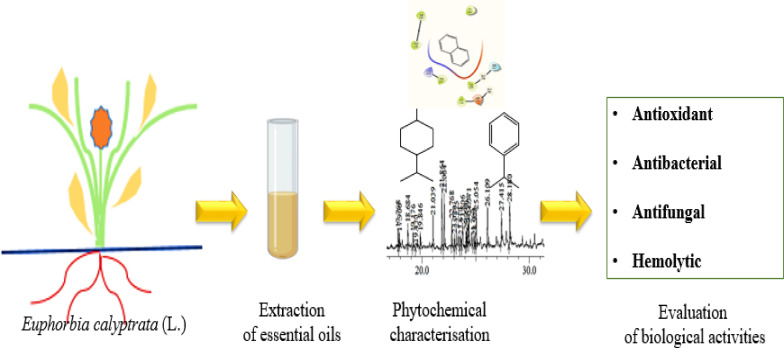

## 1 Introduction

According to the World Health Organization (WHO), in 2002, almost 80 % of the world‘s population relies on traditional medicine to access primary health care.^[1]^ This reliance on traditional medicine has led to significant economic benefits, particularly in the development of this medical approach and the use of medicinal plants to treat various ailments.[^1^, ^2^] As a result, it has become more crucial than ever to pursue research to identify the potential active compounds present in these plants.[^3^, ^4^] Morocco has a wealth of documentation on medicinal plants, emphasizing their biological, pharmacological, and phytochemical characteristics.[^5^, ^6^, ^7^] This research focuses mainly on plants used in traditional Moroccan medicine. These studies have explained the mechanism of these plants′ therapeutic action and confirmed their use in traditional medicine. However, despite numerous chemical analyses of the essential oils of many Moroccan pharmacopeia plants, little data is available on their biological properties.[^8^, ^9^, ^10^] It is important to note that essential oils are renowned for their multiple virtues, notably their antibacterial and antioxidant effects.^[11]^ The emergence of antibiotic resistance in bacteria is now a major public health concern. The problem of antibiotic resistance affects all bacterial strains and continues to worsen. Faced with this situation, it is increasingly crucial to look for alternatives, among which medicinal and aromatic plants are proving promising.[^12^, ^13^] Oxidative stress plays a central role in many pathological processes, particularly aging‐associated, such as atherosclerosis, cancer, autoimmune diseases, and conditions such as Parkinson's and Alzheimer's.^[14]^ Synthetic antioxidants, once seen as a solution in the agri‐food, cosmetics, and pharmaceutical sectors, have recently come in for sharp criticism. Indeed, there are long‐term concerns about their teratogenic, mutagenic, and carcinogenic potential.[^15^, ^16^] Plants offer an interesting alternative to synthetic antioxidants, as they contain natural antioxidants.^[15]^ The search for natural substances with antibacterial and antioxidant properties derived from plants is of great scientific importance.[^17^, ^18^] In this context, studies were undertaken to analyse the volatile compounds of *Euphorbia calyptrata* (L.), a plant among the species of the *Euphorbia* genus used in traditional Moroccan medicine.[^19^, ^20^] This plant is particularly interesting for medicine and the quest for new molecules due to its widespread use among Saharan‐Mediterranean populations to treat various infectious diseases. Examination of the chemical composition by GCMS and theoretical study of the terpenic composition extracted from *Euphorbia calyptrata* by molecular docking is the first part of the objective of this work. The second part of this work is to evaluate the biological activities (antibacterial, antifungal and antioxidant). The third part of the study evaluated the hemolytic activity to determine the in vitro toxicity of essential oils derived from a plant indigenous to the Mediterranean region, known for its use in traditional medicine.

## 2 Materials and Methods

### 2.1 Extraction and Characterization of Components within EO‐EC

The essential oil (EO) from crushed seeds was extracted using hydrodistillation for 4 hours at a temperature of 100 °C using a Clevenger apparatus. The chemical profile of the essential oil of *Euphorbia calyptrata* (EO‐EC) was determined using gas chromatography coupled with a spectrometer. For this purpose, we employed a Varian capillary column (Model: TR5‐CPSIL‐5CB) measuring 50 meters in length, 0.32 mm in diameter, and featuring a film thickness of 1.25 μm. The column underwent a gradual temperature increase, ranging from 45 to 290 °C, at a consistent rate of 4 °C per minute. The injector was consistently maintained at 280 °C, while the MS‐Polaris‐Q detector was held at 200 °C. Helium served as the carrier gas, flowing at a 1 mL/min rate. EO‐EC was injected in a volume of 1 μL after being diluted in the organic solvent hexane, employing the splitless injection technique. Electron ionization was carried out with an ionization energy of 70 eV. The ion source and interface temperatures were set to 200 °C and 350 °C, respectively. Mass scanning ranged from 30 to 650 m/z. Identification of phytochemical constituents within EO‐EC involved comparing their Kovats indices, which were calculated based on the retention times of a series of linear alkanes (C4–C29), with the reference values from standard compounds found in the Adams library and the NIST‐MS database version 2.0.

### 2.2 Evaluation of In Vitro Antioxidant Activity for EO‐EC

#### 2.2.1 DPPH Radical Scavenging Assay for EO‐EC

The antioxidant properties of EO‐EC were assessed using established methodologies. In brief, 800 μL of DPPH (0.2 mM, dissolved in methanol) were combined with 200 μL of various EO‐EC serial dilutions, ranging from 0 (utilized as the control) to 1 mg/mL. The resulting blend was incubated in darkness for 30 minutes at room temperature (R.T.). Subsequently, absorbances were measured at 517 nm, relative to a control sample comprising 800 μL of DPPH solution and methanol. Parallel positive controls employing quercetin or BHT and a blank control were prepared under identical conditions. The antioxidant activity was quantified as the absorbance inhibition (P.I.) percentage at 517 nm. The DPPH radical scavenging rate was calculated using the formula (1). The IC50 signifies the concentration, whether of EO‐EC or BHT and quercetin, necessary to decrease the presence of free radicals (DPPH) in the reaction mixture by 50 %. A lower concentration indicates a higher effectiveness of the tested products:^[21]^

(1) $$Inhibition\;\left(\%\right)={\frac{A1\;-\;A2}{A1}}^{*}100$$

Where, A1: absorbance of the control; A2: absorbance of the presence of sample.

#### 2.2.2 Β‐carotene Bleaching Assay of EO‐EC

In this assay, the antioxidant capacity of the EO‐EC is assessed by quantifying their ability to inhibit the oxidative degradation (bleaching) of β‐carotene due to the oxidation products of linoleic acid. This method follows the procedure outlined by Msaada et al., 2017.^[22]^ The β‐carotene/linoleic acid emulsion was prepared as follows: 0.5 mg of β‐carotene was initially solubilized in 1 mL of chloroform. Subsequently, 25μL of linoleic acid and 200 mg of Tween 40 were introduced into the mixture. The chloroform was completely evaporated using a rotavapor at 40 °C. Afterward, 100 mL of oxygen‐saturated distilled water were added, and the resulting emulsion was vigorously stirred. For each test, 100 or 150 μL of the extract solution or the reference antioxidant (BHT) at a concentration of 1 mg/mL were added to 2.5 mL of the previously prepared emulsion. The test tubes were then placed in a water bath and heated to 50 °C. The negative control included all the reagents except the test sample, for which an equal volume of methanol was substituted. The kinetics of emulsion decolorization were continuously monitored at 490 nm, with readings taken at 15‐minute intervals over 2 hours until the color of β‐carotene completely vanished. The antioxidant activity of EO‐EC is computed using the following formula 2:

(2) $$AA\;\left(\%\right)={\frac{AE0-AE120}{AC0-AC\;120}}^{*}100$$

AE0 represents the absorbance at 490 nm of the samples tested at t=0 minutes, while AE120 corresponds to the absorbance at 490 nm of the samples tested at t=120 minutes. AC0 represents the absorbance at 490 nm of the control at t=0 minutes, and AC120 represents the absorbance at 490 nm at t=120 minutes.

#### 2.2.3 Ferric Reducing Antioxidant Power (FRAP) Assay of EO‐EC

The ferric reduction process relies on using antioxidants to convert ferric iron into ferrous salt, forming a blue solution. Here is a summary of the procedure: In glass tubes, 200 μL of EO‐EC at various concentrations and 500 μL of 0.2 M phosphate buffer (pH 6.6) were combined, followed by the addition of 500 μL of 1 % potassium hexacyanoferrate (K_3_Fe(CN)_6_) in distilled water. The mixture was heated to 50 °C and incubated for 20 minutes in a water bath. Afterward, 500 μL of 10 % trichloroacetic acid was added, and the solution was centrifuged at 3000 rpm for 10 minutes. A 500 μL aliquot of the supernatant was then transferred to another tube, to which 500 μL of double‐distilled water (ddH2O) and 100 μL of freshly prepared 1 % FeCl_3_ in ddH2O were introduced. A blank, without including EO‐EC, was similarly prepared by substituting EO‐EC with methanol. Absorbance was measured at 594 nm against the blank, where EO‐EC was replaced with methanol, serving as the instrument calibration on a UV‐VIS spectrophotometer. Standard antioxidant solutions, either BHT or quercetin, were utilized for positive controls, and their absorbances were recorded in the same manner as for the test samples.^[23]^

### 2.3 4. Total Antioxidant Capacity (TAC) of EO‐EC

The Total Antioxidant Capacity (TAC) of EO‐EC was determined using the phosphomolybdate method, as follows: In brief, 100 μL of EO‐EC at various concentrations were combined with 1000 μL of a reagent mixture containing sulfuric acid (H_2_SO_4_), sodium phosphate (Na_2_HPO_4_), and ammonium molybdate, ensuring their concentrations fell within the ranges of 0.6 M, 28 mM, and 4 mM, respectively. The tubes were then subjected to a temperature of approximately 95 °C for 90 minutes. After cooling, absorbance was measured at 695 nm. The control consisted of 100 μL of methanol mixed with 1000 μL of the reagent mixture. Both samples and controls were incubated under identical conditions. The results are expressed in milligrams of ascorbic acid equivalents per gram (μg AAE/mg).^[24]^

### 2.4 Antimicrobial Activity

#### 2.4.1 Determination of the Zone of Inhibition on Solid Media

The disc diffusion test (diameter: 6 mm) was used to assess the antimicrobial activity of *E. calyptrata* essential oil. In brief, microbial strains, including *Staphylococcus aureus* (*S. aureus*), *Escherichia coli* (*E. coli*), *Klebsiella pneumoniae* (*K. pneumoniae*), *Pseudomonas aeruginosa* (*P. aeruginosa*), *Condida albicans* (*C. albicans*) and *Saccharomyces cerevisiae* (*S. cerevisiae*), were grown in Mueller‐Hinton (MH) and malt extract (ME) media. Fresh cultures previously grown in MH and ME media were then used to make decimal dilutions in sterile saline (NaCl: 0.1 %). The final concentration of inoculum (microbial suspension) was set at 10^6^‐10^8^ CFU/mL. Bacteria and fungi were inoculated into Petri dishes and treated with 20 μL of EO‐EC using six‐millimeter diameter discs. Conventional antibiotics, such as Streptomycin (Str) for bacterial strains and the antifungal Fluconazole (Flu) for fungal strains, the antimicrobial potency of EO‐EC was determined by calculating the zone of inhibition (mm).[^25^, ^26^]

#### 2.4.2 Determination of minimum EO‐EC Concentration (MIC)

The MICs of EO‐EC against four bacterial and two fungal strains were determined using established methods. In brief, a sterile 96‐well microplate was prepared in aseptic conditions by dispensing 100 μL of EO‐EC into the first column of the plate. Subsequently, 50 μL of MH (for bacteria) or ME (for fungi) was added. Serial dilutions were performed with a twofold dilution technique. MICs were determined via a colorimetric method employing TTC (0.2 %). This assessment took place for bacteria one‐day following injection, while for fungal strains, it was conducted two days post‐inoculation.[^13^, ^27^]

### 2.5 Molecular docking

#### 2.5.1 Protein Selection

For our computational investigation, we focused on NAD(P)H Oxidase to assess antioxidant activity.^[28]^ Additionally, we explored the antibacterial activity on *E. coli* beta‐ketoacyl‐[acyl carrier protein] synthase and *S. aureus* nucleoside diphosphate kinase^[29]^ (3). Furthermore, we investigated the antifungal potential on sterol 14‐alpha demethylase (CYP51) obtained from the pathogenic yeast *C. albicans*.^[30]^

#### 2.5.2 Protein Preparation

NAD(P)H Oxidase, *E. coli* beta‐ketoacyl‐[acyl carrier protein] synthase, *S. aureus* nucleoside diphosphate kinase, and sterol 14‐alpha demethylase (CYP51) obtained from the pathogenic yeast *C. albicans* were obtained from the Protein Data Bank (PDB) through the RCSB database (http://www.rcsb.org/) with following PDBID: 2CDU, 1FJ4, 3Q8 U, and 5FSA respectively. The structure was then imported into the Maestro interface. We followed the protein preparation protocol to ensure its suitability for computational calculations, which involved several steps. These steps included assigning bond orders, adding hydrogen atoms, creating disulfide bonds, and filling in missing side chains and loops using the Prime tool. We capped the termini to eliminate any potential interference and removed water molecules located beyond 5 Å from the hetero group.

Furthermore, we refined the chain by eliminating various heteroatoms and undesired crystallographic water molecules from the crystal structure. For accurate simulation under experimental conditions, we used the PROPKA tool to determine the protonation states of both ligands and residues. Finally, utilizing the OPLS3e force field, we performed energy minimization on the protonated structure, resulting in a lower‐energy protein structure.^[31]^

#### 2.5.3 Ligand Preparation

All essential oil compounds identified via GC‐MS were obtained from the PubChem database. These ligand structures were optimized using the LigPrep tool. To prepare the ligands at a pH of 7.0±2.0, we employed the Epik module to preserve specific chirality, desalting, and generating tautomers as required. The 2D ligand structures were then transformed into 3D structures and subjected to geometry minimization using the OPLS3e force field to attain their lowest energy conformations with necessary adjustments. These optimized ligands were subsequently utilized in the docking calculations.^[32]^

#### 2.5.4 Binding Site Identification and Molecular Docking

We utilized the receptor grid generation tool within the Glide module to identify the docking sites for the ligands within the protein structure. This grid was created to encompass the region around the co‐crystallized ligand. We intentionally excluded the co‐crystallized ligand from the minimized protein structure to prevent undue influence on the ligand‐receptor docking process. The receptor grid was generated using default parameters for Vander Waal's radius scaling factor (1 Å) and a partial charge cut‐off (0.25 Å). Flexible docking employed the Glide module, with the ligands fitting into the receptor grid at the Standard Precision (S.P) docking level. Ligands that exhibited a higher affinity for the receptor‘s active sites were identified based on their molecular interactions with both the ligand and the target protein and their docking scores (5).

#### 2.5.5 Validation of Docked Poses

To verify the precision of the docking process, we utilized the extra precision (XP) Glide docking approach. This entailed redocking the co‐crystallized ligand and previously reported inhibitors into the binding site, employing the Glide XP docking mode. The bonding interactions identified during this redocking procedure and those reported in existing literature were employed to validate the docking outcomes.

### 2.6 Hemolytic Activity

The hemolytic power of the essential oils studied was carried out in vitro at different concentrations (3, 6, 12, 25, 50 and 100 mg/mL) on a suspension of blood erythrocytes in PBS (phosphate buffered saline) at pH=7.4±0.2 [sodium chloride (137 mM), potassium chloride (2.7 mM), sodium hydrogen phosphate (8 mM), potassium dihydrogen phosphate (2 mM)].

Blood collected in heparin tubes from a healthy donor is used to prepare the erythrocyte suspension. It is centrifuged at 2500 rpm for ten minutes and, after removal of the plasma, the pellet is washed three times with PBS, then re‐suspended in the same volume of discarded plasma. The resulting erythrocyte suspension is diluted 20‐fold with PBS.

The hemolytic effect test is performed according to the method of Guo‐Xiang and Zai‐Qun.^[33]^ In the hemolysis tubes, 20 μL of EO‐EC at various concentrations were introduced into 1980 μL of the previously prepared erythrocyte suspension. Subsequently, these tubes were incubated at 37 °C for 60 minutes. After incubation, 250 μL samples were extracted from each tube and mixed with 750 μL of PBS. These mixtures were gently agitated and promptly transferred to an ice bath to halt the reaction. Subsequently, they were subjected to centrifugation at 2500 rpm for ten minutes.

Absorbance measurements were recorded at 548 nm utilizing a spectrophotometer, with a blank containing PBS as the reference. A total hemolysis tube was prepared in parallel conditions and following identical experimental protocols, consisting of 100 μL of erythrocyte suspension and 1900 μL of distilled water. A negative control tube was also created, comprising 250 μL of erythrocyte suspension and 750 μL of PBS buffer solution. The hemolysis rate for the various extracts is determined as a percentage of total hemolysis following a 60‐minute incubation period, calculated using the following formula:

(3) $$Haemolysis\;rate\;\left(\%\right)=\;\frac{Do\;S-Do\;NC}{Do\;PC}x100$$

***Do S***: Optical density of the sample (EOFP); ***Do Nc***: Negative control optical density; ***Do P.C***.: Positive control optical density.

## 3 Statistical Analysis

The results were expressed as the mean±standard deviation (SD). Statistical analyses were conducted using GraphPad Prism (version 8.0.1). The Shapiro‐Wilk test was employed for the variables to assess normality, and Levene's test was used to evaluate the homogeneity of variances. Statistical disparities among means were computed through one‐way analysis of variance (one‐way ANOVA) followed by Tukey's test for multiple comparisons. Significance levels were set at p <0.05

## 4 Results and Discussion

### 4.1 GC‐MS/MS Analysis

**Figure 1:**
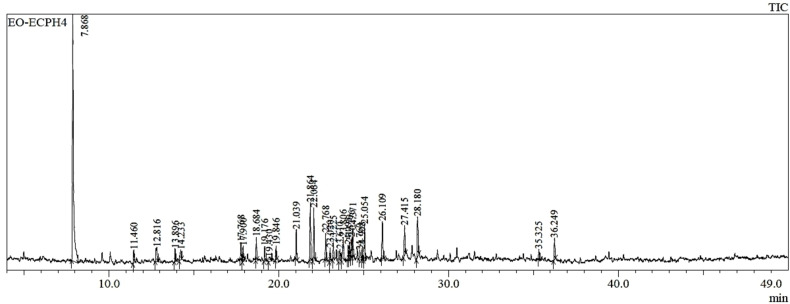
Chromatogram of EO‐EC essential oils.

Results from gas chromatography‐mass spectrometry (GC‐MS/MS) analysis of compounds present in *Euphorbia calyptrata* (L.) essential oils (Table 1 and Figure 1). 31 compounds are identified in EO‐EC using GC‐MS/MS. The table 3 include information on retention time (RT), compounds identified, retention indices (KI), chemical formulas, and peak area percentages. The list of identified compounds is diverse, covering a range of chemical classes, including monoterpenes, sesquiterpenes, diterpenes and oxygenates such as alcohols and ketones. Peak area percentages indicate the relative abundance of *alpha*‐Pinene, representing 35.96 % of the total sample composition.

**Table 1:** Chemical composition of EO‐EC essential oils analyzed by GC‐MS/MS.

Peak	R.T.	Compound	K.I.	Chemical formula (Chemical class)	Area (%)
**1**	7.868	alpha‐Pinene	939	C_10_H_16_ (MTH)	35.96
**2**	11.460	Nonanone	1090	C_9_H1_8_O (O)	1.36
**3**	12.816	Farnesane	1442	C_15_H_24_ (STH)	2.42
**4**	13.896	Heneicosane	2100	C_21_H_44_ (O)	0.92
**5**	14.233	Camphor	1146	C_10_H_16_O (MTO)	1.49
**6**	17.768	Isopulegol	1159	C_10_H_18_O (MTO)	1.48
**7**	17.900	Hexadecane	1600	C_16_H_34_ (O)	1.49
**8**	18.684	Eicosane	2000	C_20_H_42_ (DTH)	1.63
**9**	19.176	Octadecane	1800	C_18_H_38_ (O)	1.28
**10**	19.430	Terpineol	1133	C_10_H_18_O (MTO)	1.10
**11**	19.846	alpha.‐terpinyl acetate	1349	C_12_H_20_O_2_ (O)	1.12
**12**	21.039	beta‐Elemene	1390	C_15_H_24_ (STH)	2.59
**13**	21.864	Caryophyllene	1408	C_15_H_24_ (STH)	5.08
**14**	22.064	Germacrene	1485	C_15_H_24_ (STH)	4.76
**15**	22.768	beta.‐Humulene	1438	C_15_H_24_ (STH)	2.35
**16**	23.020	Geranyl linalool	1987	C_20_H_34_O (DTO)	1.12
**17**	23.225	gamma.‐Cadinene	1513	C_15_H_24_ (STH)	1.56
**18**	23.610	beta.‐Selinene	1490	C_15_H_24_ (STH)	1.36
**19**	23.806	alpha.‐Muurolene	1500	C_15_H_24_ (STH)	2.99
**20**	24.093	Shyobunol	1689	C_15_H_26_O (STO)	1.11
**21**	24.179	Naphthalene	1181	C_10_H_18_ (MTH)	1.40
**22**	24.296	gamma.‐Cadinene	1513	C_15_H_24_ (STH)	1.66
**23**	24.371	cis‐Calamenene	1529	C_15_H_22_ (STH)	2.44
**24**	24.760	alpha.‐terpinene	1017	C_10_H16 (MTH)	1.50
**25**	24.908	cis‐calamenene	1529	C_15_H_22_ (STH)	1.10
**26**	25.054	Elemol	1549	C_15_H_26_O (STO)	3.61
**27**	26.109	Caryophyllene	1669	C_15_H_24_O (STO)	4.38
**28**	27.415	Cadinol	1640	C_15_H_26_O (STO)	2.98
**29**	28.108	alpha‐Muurolol	1642	C_15_H_26_O (STO)	4.76
**30**	35.275	Bisabolene	1531	C_15_H_24_ (STH)	0.91
**31**	36.249	Manool oxide	2010	C_20_H_34_O (DTO)	2.02
			

		Chemical class			
		Monoterpene hydrocarbons (MTH)			38.86
		Monoterpene Oxygenated (MTO)			4.07
		Sesquiterpene hydrocarbons (STH)			29.22
		Sesquiterpene Oxygenated (STO)			16.84
		Diterpene hydrocarbons (DTH)			1.63
		Diterpene Oxygenated (DTO)			3.14
		Others (O)			6.17
		Total (%)			99.93

The analysis of the essential oil has revealed a diversity of chemical compounds, each belonging to a specific category based on its structure. Monoterpene hydrocarbons (MTH) constitute the largest portion of the total composition, accounting for 38.86 % of the essential oil. Monoterpene oxygenated compounds (MTO) are also present, albeit to a lesser extent, representing 4.07 % of the composition. Sesquiterpene hydrocarbons (STH) form a substantial part at 29.22 %, while oxygenated sesquiterpenes (STO) make a notable contribution of 16.84 %. Diterpene hydrocarbons (DTH) and oxygenated diterpenes (DTO) are in smaller quantities, accounting for 1.63 % and 3.14 % of the total composition, respectively. Lastly, an Others (O) category encompasses compounds that do not fit clearly into the previous categories, contributing 6.17 % to the overall composition of the essential oil. This distribution of chemical classes provides insight into the essential oil‘s complexity and its constituents′ diversity (Table 1).

The compounds identified may play an important role in the pharmacological properties and chemical characteristics of *Euphorbia calyptrata*. Some of the compounds identified, such as *alpha*‐pinene, Caryophyllene, and others, are known to have potential pharmacological properties due to their specific chemical structures.[^34^, ^35^] These compounds could contribute to the pharmacological activities of *Euphorbia calyptrata* (L.).^[19]^

The distribution of chemical classes within an essential oil is significant in pharmacology because each class of compounds possesses specific pharmacological properties. For instance, monoterpene hydrocarbons may exhibit anti‐inflammatory and antimicrobial properties, while oxygenated monoterpenes are known for their antiseptic effects. Sesquiterpene hydrocarbons can assist in reducing inflammation, sesquiterpene oxygenated compounds have immunostimulant properties, and diterpenes can have various effects, including anticancer properties. Understanding the chemical composition of an essential oil is crucial for determining its pharmacological applications safely and effectively.[^36^, ^37^, ^38^, ^39^, ^40^, ^41^]

### 4.2 Antioxidant Activity

The data in Table 2 represent the results of the antioxidant activity of OE‐EC at different concentrations, measured as a percentage of DPPH inhibition, with the associated standard deviations. At a concentration of 50 μg/ml OE‐EC, the antioxidant activity was 45.08 %±0.20 %. This means that at this concentration, OE‐EC reduces DPPH inhibition by 45.08 % on average, with some variability in the measurements represented by the standard deviation of 0.20 %.

**Table 2:** Antiradical activity and IC_50_ of essential oil derived from EO‐EC, BHT, and quercetin by DPPH test.

	50 μg/mL	100 μg/mL	200 μg/mL	400 μg/mL	800 μg/mL	1000 μg/mL	IC_50_ (μg/mL)
EO‐EC	45.08±0.20	69.51±0.71	79.89±1.02	85.19±0.92	89.74±0.65	96.35±0.31	67.28±1.51
BHT	61.46±0.15	76.48±0.53	88.10±0.92	90.07±0.48	94.61±0.59	90.45±0.90	40.58±0.95
Quercetin	41.97±0.09	59.60±0.41	70.02±0.83	81.42±0.39	89.83±0.66	93.64±0.81	72.81±1.24

Antioxidant activity increased with the concentration of EO‐EC. At a concentration of 1000 μg/ml, antioxidant activity reached 96.35 %±0.31 %, indicating a high antioxidant capacity at this specific concentration. These data suggest that EO‐EC has a dose‐dependent antioxidant activity, meaning that the higher the concentration of EO‐EC, the higher its antioxidant capacity. This information can be valuable in assessing the potential of EO‐EC as an antioxidant, particularly at higher concentrations. The antioxidant activity of EO‐EC increases progressively with concentration. At 1000 μg/mL, EO‐EC reaches an activity of 96.35 %, which is comparable to the activity of BHT at this concentration. Quercetin, although exhibiting lower antioxidant activity than BHT at most concentrations, also showed increased activity with increasing concentration. At 1000 μg/mL, quercetin achieves 93.64 % activity.

IC_50_ results for BHT, EO‐EC, and quercetin are presented in the Table 2. BHT has the lowest IC_50_ of the three substances, the most effective at inhibiting antioxidant activity. A lower concentration of BHT (40.58 μg/mL) is required to reduce antioxidant activity by 50 % compared with the concentrations used in the study. EO‐EC has an IC_50_ of 67.28 μg/mL, indicating that a slightly higher concentration of EO‐EC than BHT is required to achieve the same level of 50 % antioxidant inhibition. Quercetin has the highest IC_50_ of 72.81 μg/mL, suggesting it is less effective at inhibiting antioxidant activity than BHT and EO‐EC (Table 2). An even higher concentration of quercetin is required to achieve the same inhibitory effect. These data provide a valuable indication of the relative ability of the substances to neutralize free radicals in the DPPH assay.

The choice of the DPPH test to assess antioxidant activity is relevant, as it is widely recognized in scientific research for its sensitivity to the ability of compounds to neutralize free radicals. This measurement method is commonly used to assess the antioxidant capacity of plant extracts.[^42^, ^43^] In addition to the ability to neutralize free radicals, EO‐EC has also shown an effect on free radical chains. This suggests that these essential oils can interrupt free radical chain reactions, which may have beneficial consequences for preventing oxidative stress.[^15^, ^44^] The presence of powerful antioxidant compounds in EO‐EC opens opportunities for developing pharmaceutical and cosmetic products.[^14^, ^45^] These products could potentially help prevent or treat problems linked to oxidative stress, such as degenerative diseases, dermatological conditions, and signs of premature aging.[^46^, ^47^]

EC_50_ results for EO‐EC, BHT, and quercetin measured using the FRAP method are shown in Table 3. The EC_50_ is an important measure of antioxidant activity, indicating the concentration required to achieve a 50 % reduction in ferric ions in the FRAP assay. The EC_50_ of EO‐EC is 80.25 μg/mL±1.42 μg/mL (Table 3). BHT requires a higher concentration (120.46 μg/mL±2.03 μg/mL) than EO‐EC to achieve a 50 % reduction in ferric ions in the FRAP test. Quercetin had the lowest EC_50_ of the three substances tested (74.48 μg/mL±1.40 μg/mL), which was the most effective at reducing ferric ions in the FRAP assay (Table 3). The EC_50_ results in the FRAP assay confirm that EO‐EC has significant antioxidant capacity. Its EC_50_ is lower than that of BHT, indicating that EO‐EC is more effective than BHT in reducing ferric ions. This suggests that EO‐EC could be a potentially useful antioxidant, making it attractive as a natural alternative to synthetic antioxidants such as BHT.

**Table 3:** Antioxidant activity of EO‐EC essential oils by FRAP and Beta‐carotene tests.

	FRAP	*Beta*‐carotene
	EC_50_ (μg/mL)	Inhibition (%)
EO‐EC	80.25.08±1.42	94.83±2.11
BHT	120.46±2.03	96.42±1.35
Quercetin	74.48±1.40	66.55±2.08

The results of this study firmly established a correlation between antioxidant activity, measured using the FRAP test, and the presence of antioxidant compounds in *Euphorbia calyptrata* (L.) essential oils. These antioxidant compounds, such as alpha‐Pinene, Caryophyllene, and alpha‐Muurolol (as the majority compound), play an essential role in the ability of this plant‘s essential oils to neutralize free radicals.[^42^, ^48^] Identifying alpha‐Pinene, Caryophyllene, and alpha‐Muurolol as the main antioxidant compounds in *Euphorbia calyptrata* (L.) essential oils is essential. It is important to discuss in detail the ability of these compounds to neutralize free radicals, explaining how they act at the molecular level to protect cells and tissues from oxidative damage.[^16^, ^42^, ^49^]

The results for inhibition (%) of beta‐carotene by EO‐EC, BHT, and quercetin are shown in Table 3. Inhibition of beta‐carotene by EO‐EC was 94.83±2.11 %. BHT showed an even higher inhibition of beta‐carotene oxidation of 86.42±1.35 %. Quercetin also showed a significant ability to inhibit beta‐carotene oxidation by 66.55±2.08 %. In this beta‐carotene test, EO‐EC proved to be the most effective antioxidant of the three substances tested, closely followed by BHT and then quercetin.

Essential oils from *Euphorbia calyptrata* (L.) contain antioxidants and have also been shown to affect free radical chains. It is necessary to discuss how these essential oils interrupt these reaction chains, which can benefit health, notably by preventing premature aging and diseases linked to oxidative stress. The presence of powerful antioxidants in *Euphorbia calyptrata* (L.) essential oils opens potential applications in medicine and cosmetics. These oils could be exploited to develop pharmaceutical or skin care products aimed at preventing or treating problems linked to oxidative stress,[^15^, ^42^] such as cardiovascular disease,^[14]^ cancer or skin aging.[^50^, ^51^]

The results of the TAC (Total Antioxidant Capacity) test measuring the antioxidant activity of EO‐EC, BHT, and quercetin at different concentrations (100 μg/mL, 250 μg/mL, 500 μg/mL, 1000 μg/mL) are presented in Table 4. At 100 μg/mL, EO‐EC has an antioxidant activity of 80.25±1.42. This antioxidant activity increases with concentration, reaching 194.83±5.11 μg EAA/mg at 250 μg/mL, 785.39±2.58 μg EAA/mg at 500 μg/mL, and 985.07±0.70 μg EAA/mg at 1000 μg/mL.

**Table 4:** Antioxidant activity of EO‐EC essential oils by TAC assay.

	100 μg/mL	250 μg/mL	500 μg/mL	1000 μg/mL
EO‐EC	80.25.08±1.42	194.83±5.11	785.39±2.58	985.07±0.70
BHT	120.46±2.03	360.44±7.12	805.95±0.92	992.55±0.98
Quercetin	74.48±1.40	156.55±3.45	583.66±5.81	886.98±1.55

Antioxidant power by the phosphomolybdate method, at low concentration (100 mg/mL), revealed that BHT was the most effective, followed by EO‐EC and quercetin, with antiradical power of the order of 120.46±2.03, 80.25.08±1.42 and 74.48±1.40 μg EAA/mg, respectively. The antioxidant activity of BHT also increased with concentration, reaching 360.44±7.12 μg EAA/mg at 250 μg/mL, 805.95±0.92 μg EAA/mg at 500 μg/mL, and 992.55±0.98 μg EAA/mg at 1000 μg/mL. Quercetin showed lower antioxidant activity than EO‐EC and BHT at all concentrations tested. The antioxidant activity of quercetin also increased with concentration, reaching 156.55±3.45 μg EAA/mg at 250 μg/mL, 583.66±5.81 μg EAA/mg at 500 μg/mL, and 886.98±1.55 μg EAA/mg at 1000 μg/mL. The results show that EO‐EC, BHT, and quercetin have significant antioxidant activity. EO‐EC and BHT show similar antioxidant activities at all concentrations tested, with comparable activity values. EO‐EC and BHT show similar antioxidant activities, suggesting that EO‐EC has an antioxidant potential comparable to BHT, a commonly used synthetic antioxidant.

These results indicate that EO‐EC could be a promising candidate as a natural antioxidant. However, further research is needed to assess its efficacy in various contexts and understand its mechanisms of antioxidant action.^[52]^ The results of these studies provide solid evidence of the antioxidant activity of *Euphorbia calyptrata* (L.) essential oils and of the correlation between this activity and the presence of antioxidant compounds such as alpha‐Pinene, Caryophyllene, and alpha‐Muurolol.[^46^, ^53^] These discoveries have great potential for the pharmaceutical industry, cosmetics, and medical research while raising exciting questions for future research.

### 4.3 Antimicrobial Activity

Table 5 displays the inhibitory zone diameters and MICs of *E. calyptrata* essential oils against the bacterial and fungal strains tested. *E. calyptrata* essential oils have been shown to have very significant inhibitory activity against the bacterial strains tested.

**Table 5:** Inhibitory zone diameters and MICs of *E. calyptrata* essential oils.

		***Antibacterial activity***				***Antifungal activity***	
		*E. coli*	*K. pneumoniae*	*P. aeruginosa*	*S. aureus*	*S. cerevisiae*	*C. albicans*
EOEC	Di (mm)	10.28±0.57	15.48±0.63	25.80±0.85	19.04±0.69	31.48±0.59	34.21±0.80
	MIC (μg/mL)	24.91±1.52	22.62±0.74	10.27±0.94	10.85±0.38	9.32±0.44	19.08±0.30
Str	Di (mm)	14.20±0.98	0.0±0.0	24.61±0.74	0.0±0.0	N.T.	N.T.
	MIC (mg/mL)	11.70±0.84	0.0±0.0	9.29±0.48	0.0±0.0	NT	NT
Fluc	Di (mm)	N.T.	N.T.	NT	NT	20.59±1.00	26.08±1.05
	MIC (mg/mL)	N.T.	N.T.	NT	NT	0.26±0.07	0.41±0.09

Not tested (NT), Streptomycin (Str), Inhibition diameter (Di), Fluconazole (Flu), Staphylococcus aureus (S. aureus), Escherichia coli (E. coli), Klebsiella pneumoniae (K. pneumoniae), Pseudomonas aeruginosa (P. aeruginosa), Condida albicans (C. albicans), Saccharomyces cerevisiae (S. cerevisiae).

The EO of this plant was found to have a greater antibacterial effect against Gram (+) bacteria than against Gram (‐) bacteria, such that it was found to have an inhibitory activity of the order of 25. 80±0.85 and 15.48±0.63 mm on solid medium, and a MIC of the order of 10.27±0.94 22.62±0.74 μg/mL on liquid medium, for *P. aeruginosa* and *K. pneumonia*, respectively.

Essential oils of *E. calyptrata* are more antifungal than Fluconazole against *S. cerevisiae* and *C. albican*. This activity is due to the chemistry of the essential oils, which are high in alpha‐Pinene, which has an antimicrobial effect. This molecule has been shown in studies to have an antibacterial effect against various Gram‐positive bacteria, and alpha‐Pinene has an inhibitory effect against *S. aureus* with a MIC of 256 μg /mL.^[54]^ Among the majority of EO (5.08 %) beta‐caryophyllene compounds is a bicyclic sesquiterpene with a rare 1,1‐dimethylcyclobutane ring found in various plants′ essential oils. The antibacterial activity of beta‐caryophyllene has been reported for several bacterial strains, enhancing their antimicrobial potential. Previous research has found that beta‐caryophyllene has antifungal activity. These findings suggest that the antifungal activity observed in our study may result from a combined effect of these EO sesquiterpenes.[^55^, ^56^]

### 4.4 Molecular Docking

Regarding antioxidant activity, the compounds Naphthalene, Shyobunol, and Manool oxide displayed the highest activity against NADPH oxidase, exhibiting Glide G‐scores of −5.294, −5.218, and −5.161 kcal/mol, respectively (Table 6). For antibacterial activity against *E .coli* beta‐ketoacyl‐[acyl carrier protein] synthase, the most potent molecules were cis‐Calamenene, alpha.‐Muurolene, and Terpineol, with Glide G‐scores of −6.804, −6.424, and −6.313 kcal/mol, respectively. On the other hand, within the active site of *Staphylococcus aureus* nucleoside diphosphate kinase, Cadinol, alpha.‐Terpinene, and Naphthalene displayed the highest activity, with Glide scores of −5.714, −5.482, and −5.375 kcal/mol (Table 6). Regarding antifungal activity against sterol 14‐alpha demethylase (CYP51) obtained from the pathogenic yeast *Candida albicans*, the most active molecules were Manool oxide, Cadinol, and cis‐Calamenene, with Glide scores of −8.478, −7.319, and −7.317 kcal/mol (Table 6).

**Table 6:** Docking results of ligands inactive.

Title	2CDU ‐ minimized		1FJ4 ‐ minimized		3Q8 U ‐ minimized		5FSA ‐ minimized	
	glide gscore	glide energy	glide gscore	glide energy	glide gscore	glide energy	glide gscore	glide energy
alpha.‐Muurolene	−4.808	−18.564	−6.424	−23.553	−4.556	−19.163	−7.264	−26.328
alpha.‐terpinene	−4.803	−19.186	−5.84	−21.708	−5.482	−16.816	−5.655	−19.495
alpha.‐Terpinyl acetate	−4.398	−25.258	−6.075	−28.229	−4.552	−22.872	−6.018	−27.71
alpha‐Muurolol	−3.366	−20.156	−4.062	−18.983	−4.136	−17.195	−7.2	−24.957
alpha‐Pinene	−4.091	−10.067	−5.663	−17.815	−4.136	−13.701	−4.959	−18.21
beta.‐Humulene	−4.513	−19.728	−3.538	−17.513	−4.371	−14.436	−6.883	−26.087
beta.‐Selinene	−4.492	−13.148	−3.666	−18.051	−3.862	−17.287	−6.038	−20.089
beta‐Elemene	−2.688	−13.811	−5.361	−24.355	−3.936	−20.854	−5.606	−22.782
Bisabolene	−3.326	−19.113	−5.535	−27.586	−4.908	−22.328	−5.741	−25.249
Cadinol	−5.143	−14.964	−6.067	−20.675	−5.714	−21.687	−7.319	−25.692
Camphor	−3.845	−17.537	−5.948	−20.628	−4.572	−10.817	−5.48	−22.203
Caryophyllene	−4.343	−11.633	−5.064	−11.924	−4.157	−7.755	−7.025	−24.471
cis‐Calamenene	−4.655	−21.628	−6.804	−24.922	−5.046	−18.287	−7.317	−27.383
Eicosane	1.065	−29.948	–	–	1.869	−26.088	−1.24	−33.377
Elemol	−3.419	−13.436	−4.584	−18.491	−3.059	−14.237	−6.188	−24.898
Farnesane	0.112	−22.814	−1.42	−25.007	−0.118	−18.572	−2.124	−27.743
gamma.‐Cadinene	−4.845	−18.674	−5.948	−21.84	−5.202	−17.818	−6.911	−26.044
Geranyl linalool	−2.757	−33.286	−2.674	−33.804	−2.001	−32.901	−3.803	−35.638
Germacrene	−4.152	−16.079	−4.405	−18.719	−4.192	−18.685	−6.19	−22.648
Heneicosane	1.081	−30.407	–	–	2.75	−23.946	−1.633	−34.421
Hexadecane	–	–	0.393	−25.787	2.83	−23.553	0.908	−28.978
Isopulegol	−4.43	−15.828	−5.732	−21.048	−5.264	−18.502	−5.526	−30.346
Manool oxide	−5.161	−13.553	−5.362	−27.466	−3.289	−14.12	−8.478	−42.546
Naphthalene	−5.294	−20.051	−5.982	−20.807	−5.375	−16.385	−6.389	−19.733
Nonanone	−2.353	−20.626	−3.465	−26.143	−2.525	−20.342	−3.441	−23.385
Octadecane	0.498	−31.12	0.283	−28.461	3.25	−18.85	−1.06	−30.226
Shyobunol	−5.218	−18.568	−5.951	−23.382	−4.672	−21.584	−7.129	−23.435
Terpineol	−4.364	−17.396	−6.313	−20.894	−5.119	−19.017	−5.921	−25.224

**Figure 2:**
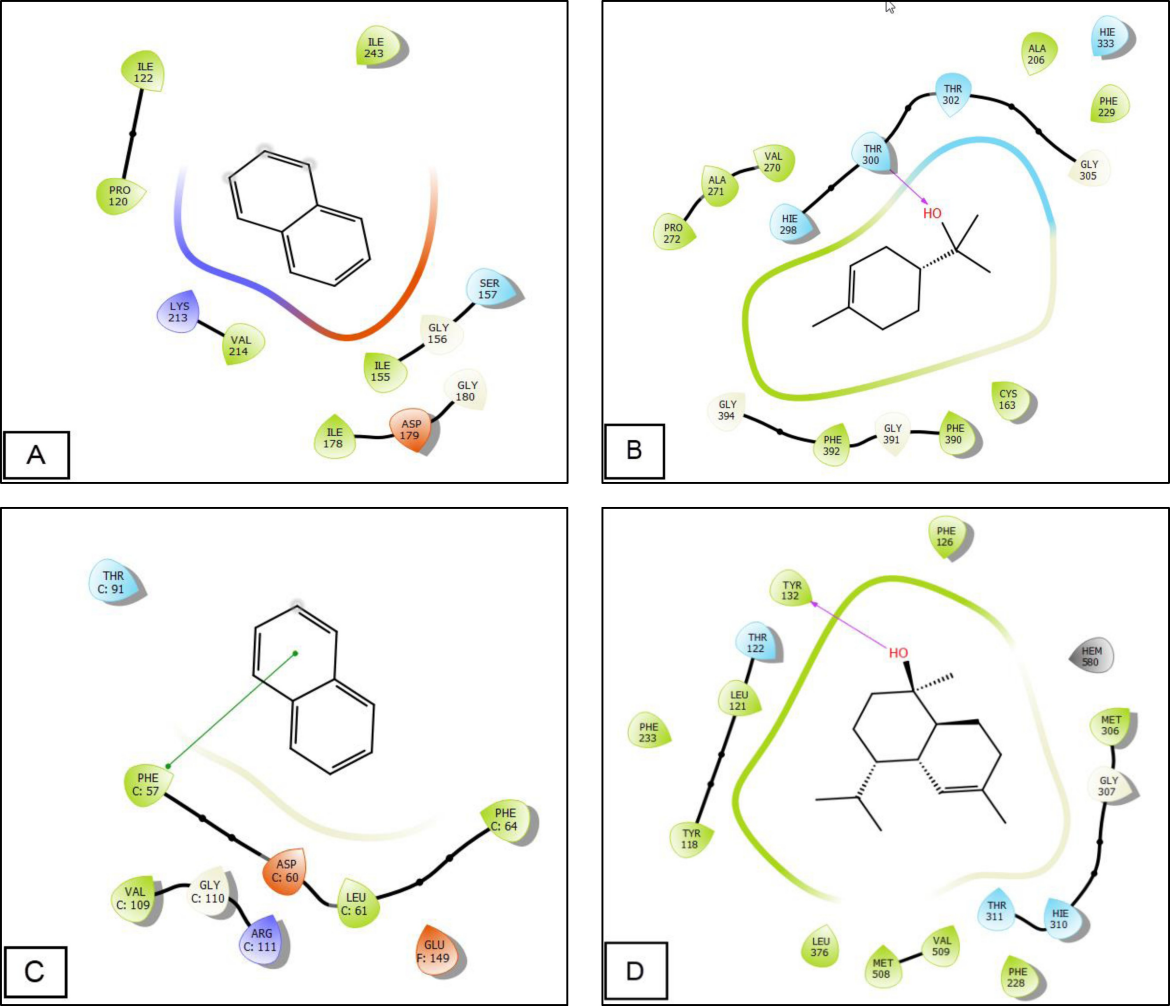
2D Diagrams of ligand interactions with the active site. (a) and (c) Naphthalene interactions with the active sites of NADPH oxidase and *Staphylococcus aureus* nucleoside diphosphate kinase; (b) Terpineol interactions with the active sites of *E. coli* beta‐ketoacyl‐[acyl carrier protein] synthase; (d): Cadinol interactions with the active sites of sterol 14‐alpha demethylase (CYP51) obtained from the pathogenic yeast *Candida albicans*.

**Figure 3:**
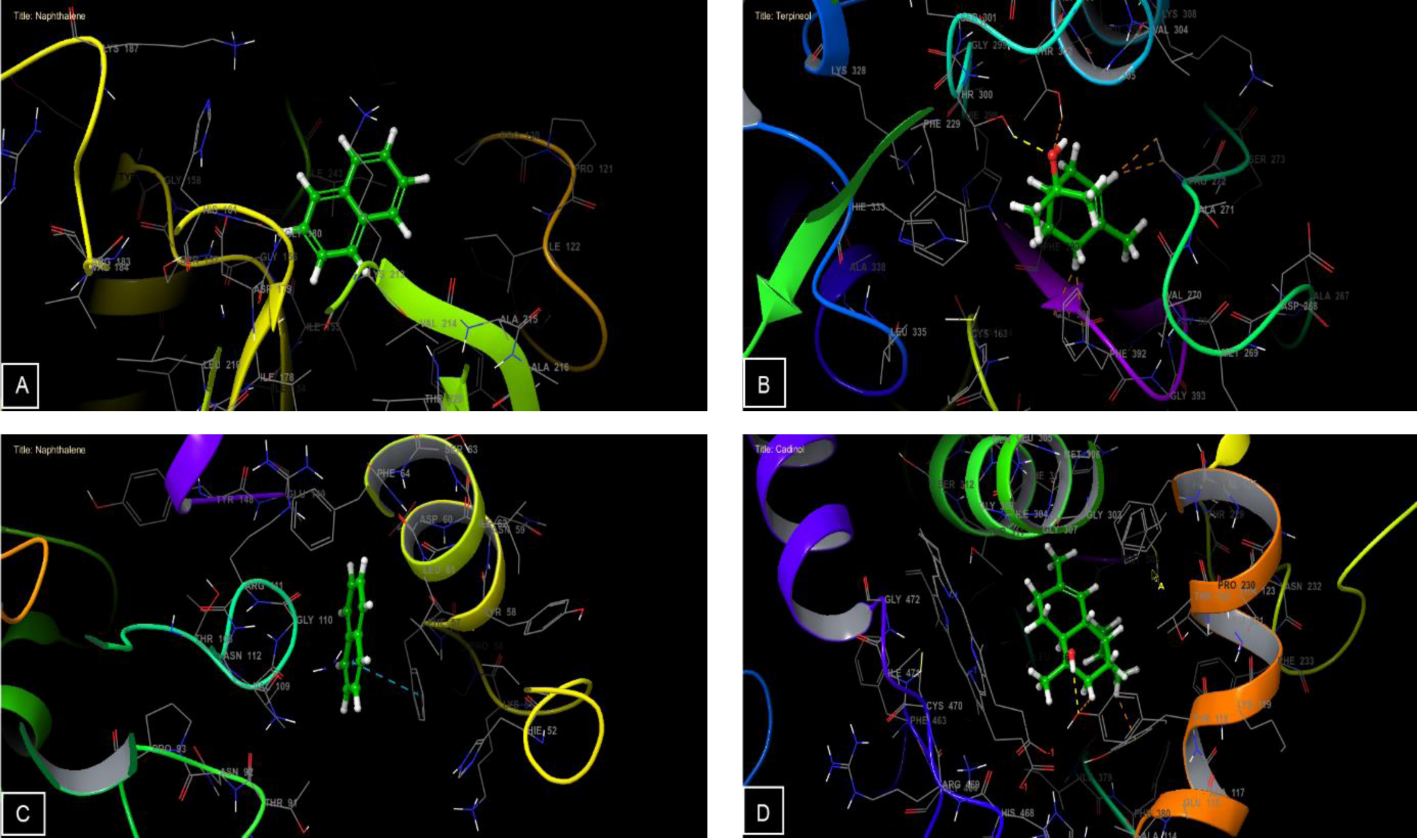
3D Diagrams of ligand interactions with the active site. (a) and (c) Naphthalene interactions with the active sites of NADPH oxidase and *Staphylococcus aureus* nucleoside diphosphate kinase; (b) Terpineol interactions with the active sites of *E. coli* beta‐ketoacyl‐[acyl carrier protein] synthase; (d): Cadinol interactions with the active sites of sterol 14‐alpha demethylase (CYP51) obtained from the pathogenic yeast *Candida albicans*.

The docking analysis of Terpineol within the active site of *E. coli* beta‐ketoacyl‐[acyl carrier protein] synthase revealed the creation of a lone hydrogen bond with residue THR 300 (Figure 2 B and 3 B). Conversely, Naphthalene formed a single π‐π stacking interaction with PHE 57 residue within the active site of *Staphylococcus aureus* nucleoside diphosphate kinase (Figure 2 C and 3C). In the case of Cadinol, when docked into the active site of sterol 14‐alpha demethylase (CYP51) isolated from the pathogenic yeast *Candida albicans*, it exhibited the formation of a sole hydrogen bond with residue TYR 132 (Figure 2D and 3D).

### 4.5 Hemolytic Activity

Results relating to the inhibition of hemolytic activity at different concentrations are presented in Table 7. Hemolytic activity generally refers to the ability of a substance to prevent or reduce the destruction of red blood cells (hemolysis). The values shown are the percentage inhibition of hemolytic activity at different concentrations.

**Table 7:** Hemolytic Effect of EO‐EC.

	3 μg/mL	6 μg/mL	12 μg/mL	25 μg/mL	50 μg/mL	100 μg/mL
Inhibition (%)	1.57±0.21	3.68±0.19	5.81±0.48	7.46±0.24	8.04±0.62	9.42±0.33

These results indicate that, as the concentration of the substance increases, there is a dose‐dependent increase in the inhibition of hemolytic activity. In other words, higher concentrations of the substance lead to greater red blood cell destruction inhibition. Values expressed as percent inhibition of hemolytic activity indicate the extent to which the substance tested is capable of counteracting hemolysis.^[57]^ The higher the inhibition percentage, the more effectively the substance protects red blood cells from destruction. This can be crucial in contexts such as drug development or research into the toxic effects of chemicals.^[33]^

Hemolytic activity and inhibition percentages are important parameters in medical research, toxicology and related fields, as they enable us to assess how substances affect the fragility of red blood cells and may have important implications for human health.^[58]^ In toxicology, hemolytic activity can be used to assess the potential harmful effects of certain substances on red blood cells. Strong inhibition of hemolytic activity may indicate that the substance protects against damage to red blood cells.^[5]^ The measurement of hemolytic activity is often used in medical research to assess the efficacy of drugs or chemical compounds in treating diseases associated with hemolysis, such as hemolytic anemias. Substances inhibiting hemolysis may have therapeutic potential for these affections.^[33]^

## 5 Conclusions

In conclusion, this study looked at essential oils derived from the plant *Euphorbia calyptrata* (L.), a species deeply rooted in traditional Moroccan medicine and widespread in the Saharan‐Mediterranean region. The main objective was to examine their chemical composition and assess their antibacterial and antioxidant properties. The results of this study provide crucial information on the chemical constituents present in the essential oils of this botanical specimen, as well as their potential roles as antibacterial and antioxidant agents. These data have significant implications for traditional medicinal practices and the ongoing search for new therapeutic compounds. Consequently, this study enhances our understanding of the plausible medicinal properties inherent in *Euphorbia calyptrata* (L.), laying the foundation for future research efforts aimed at exploiting these compounds to combat infectious diseases and improve general well‐being.

## Author Contributions

Conceptualization, writing the original draft, reviewing and editing: Fatima El Kamari, Otmane zouirech, Amira Metouekel, Mohammed Bouslamti, Imane Maliki. Formal analysis, investigations, funding acquisition, reviewing, and editing: Abdelfattah El Moussaoui, Mohamed Chebaibi, Mohamed Taibi, Khalid S. Almaary, Hiba‐Allah Nafidi. Resources, data validation, data curation, and supervision: Mohammed Bourhia, Musaab Dauelbait, Abdelfattah Abdellaoui.

## Conflict of interests

Authors declare no conflict of interest.

6

## Data Availability

The data that support the findings of this study are available from the corresponding author upon reasonable request.
